# A New Name, If Not a New Disease

**Published:** 1890-08-16

**Authors:** 


### The Practitioner's Mirror and Retrospect.

[*The Editor will be glad to receive offers of co-operation and contributions from members of the profession. There is no doubt that much valuable material full of interest and instruction is at present lost to science. This is largely so in the case of* (1) *the younger and less-known members of the hospital staff* (2) *those connected with cottage hospitals, and* (3) *the great body of medical practitioners not attached to any institution. The co-operation of all is cordially invited to enable the Editor to make* The Hospital *of the utmost possible use and value to the profession. Specialists willing to review books on their special subjects are invited to communicate this fact at once. All letters should be addressed to* The Editor, The Lodge, Porchester Square, London, W.]

### MEDICINE.

### A NEW NAME, IF NOT A NEW DISEASE.

Dr. George Thin has recently published an article (*British Medical Journal*) headed "Psilosis (Linguæ et mucosæ intestini)," a malady which has been termed "Sprue," especially by medical men in Batavia, where it is common. Dr. Thin says that, briefly summarised, psilosis may be described as a chronic affection of the intestinal tract, unaccompanied by fever or any abnormality in the urine, with a remarkable and unusual disposition to an affection of the tongue and buccal mucous membrane. There is no blood nor mucous in the stools, and there is no straining. As a rule the diarrhœa is not acute or severe, although it may be so paroxysmally. The motions are either of a pale straw, yellowish colour, frothy, quite unformed, in the first stages being chiefly passed in the early morning, or they consist of a greyish white pultaceous mass covered with froth. There is no jaundice nor bile in the urine. In many cases the symptoms in the mouth are particularly troublesome. The tongue or mucous membrane of the mouth may become the seat of an eruption, which is almost herpetic at the outset, or the whole tongue, or patches of it, may simply become red and bare. It is to express this condition, bareness, or rawness of the mucous membrane that Dr. Thin proposes the term psilosis, as the whole mucous membrane of the intestinal tract is probably more or less similarly affected. Dr. Thin then details several cases coming under his notice, illustrative of the above symptoms. Now, some time ago, Sir Joseph Fayrer pointed out the resemblance between the malady called "Sprue," and the malady known in India as hill diarrhœa (because it is most prevalent on the hills), or diarrhœa alba. Turning to the most recent author on Indian Diseases, Sir William Moore, we find chronic tropical diarrhœa thus described: "A more or less painless diarrhœa, of light grey, sometimes almost white, frothy, offensive, and fatty stools. At first, this chiefly occurs in the morning. Although the diarrhœa is generally painless the person is much troubled with rumbling in the bowels, and with flatus which may have a rotten-egg flavour. Although little or no bile is present, it is still not found in the urine. The skin although often bronzed or even sallow, is not jaundiced, but it is dry and harsh, and loose from the absorption of fat. In severe cases the tongue is red and glazed, and there may be apthous spots in the mouth. There is increasing emaciation and languor." It is therefore evident that the malady described by Sir William Moore as "chronic tropical diarrhœa," is the malady now written of by Dr. Thin under the heading "Psilosis, linguæ et mucosæ intestini." There are also other points of resemblance. Moore regards the malady as hepatic in the first instance, and due to atmospheric changes or chill; and Dr. Thin commences the details of one of his cases with the observation that "in the winter of 1877 the patient was severely chilled by exposure, &c." Sir W. Moore says that after death the intestines have been found attenuated, the mucous coat and glands also the mesenteric glands being degenerated and atrophied with fatty or lardaceous deposits. The liver and spleen have been found smaller than natural without alteration of structure but sometimes with smaller deposits. And Dr. Thin states, as the result of a *post mortem* examination in one of his cases. "The walls of the duodenum were thin and lined with a layer of mucoid material. . . . The jejunum and ileum showed similar appearances. . . . Peyers patches could not be made out. . . . The liver was soft and friable, but without change of structure." Dr. Thin is disposed to consider that the pathological changes suggest some morbid agent acting from the free surface, leading to a chronic and low form of congestion of the superficial blood vessels, with consequent gradual destruction of the epithelial structures. But Sir William Moore gives a different view. He states the condition assimilates to that described by Dr. D. Cunningham as occurring in the intestines of famine-stricken people, and, he says, "it is most probable that these appearances are consequent on the disease and are not the original cause of the disease." As in the famine-stricken they result from want of nutrition, which want of nutrition is caused by diarrhœa in the one case, and by famine in the other. "A very few days of profuse diarrhœa, with its attendant loss of appetite, and drain on the system is equivalent to famine." The explanation of diarrhœa alba is believed by Sir William Moore to be torpor of the secreting cells of the liver, the result of chill. For diarrhœa alba, or torpor of the liver, sometimes presents within a few hours of a person passing from the heated Indian plains into the comparatively cold air of the Indian mountains; or when a person, journeying from the tropics, viâ Egypt, enters the colder atmosphere of the Gulf of Suez, or the still colder air of the Mediterranean. Why the liver should be so often affected is probably explainable by the irritable condition of the organ in Eastern climates rendering it more susceptible. But the liver is not always affected. A similar condition of torpor of other organs may result from cold. The bladder may become powerless to act, or a limb may become temporarily numbed and powerless. It is the absence of bile which causes the diarrhœa in the first instance. The fact that diarrhœa may be caused by both excess and deficiency of bile is not paradoxical. Anything irritating the intestines will cause diarrhœa. There are, as the results of absence of bile, imperfect digestion, non-neutralization of irritating acids, and the loss of the antiseptic properties of the bile. Jaundice and head symptoms not occurring, there being no bile in the urine and little in the motions, leads to the conclusion that little or no bile is formed. One of the constituents of the bile may probably pass off in the sulphuretted hydrogen eructations. Mon. Robin, some time back, stated that *acholia* is characterized by the elimination of incompletely oxidised sulphur in the urine. But in *diarrhœa alba* it probably eliminates otherwise. Lastly, Sir William Moore regards the aphthous condition of mouth and the condition of the bowels as secondary results. If the term psilosis is to be adopted we shall merely have the malady designated by a prominent symptom, which, however, is not always present, a symptom, in fact, which varies much, both in degree and the time at which it presents. It is satisfactory to add, that with the exception of name and causation, both the authors we have quoted are pretty much in accord, especially as regards treatment. Both agree that it should be prompt, and both agree with Sir Joseph Fayrer, that in severe cases a milk diet is usually most beneficial. Sir William Moore, however, adds a caution. He says the anterior condition must be studied. The possibility of a scurvy taint should never be ignored, which may exist in a latent condition, and one of the symptoms of which may actually be light coloured diarrhœa stools. "If," he says, "the patient has been in a locality where fresh vegetables were scarce, or, if he has fallen into the Anglo-Indian habit of eating vegetables scantily, he should have anti-scorbutics, even although there are no visible indications of scurvy."

